# Internet Addiction and Psychological Distress: Can Social Networking Site Addiction Affect Body Uneasiness Across Gender? A Mediation Model

**DOI:** 10.5964/ejop.10273

**Published:** 2024-02-29

**Authors:** Rossella Bottaro, Giusy Danila Valenti, Palmira Faraci

**Affiliations:** 1 Department of Human and Social Sciences, University of Enna “Kore”, Enna, Italy; 2 Department of Psychological, Educational Sciences and Human Movement, University of Palermo, Palermo, Italy; University of South Wales, Cardiff, United Kingdom

**Keywords:** Internet addiction, social networking sites, body uneasiness, self-esteem, psychological distress

## Abstract

**Introduction:**

The Internet, with its unlimited information, revolutionary communication capabilities, and innovative potential to expand knowledge, is ubiquitous throughout the world, but it also has significant implications for users’ mental health. Given the not yet clearly defined and distinguishable nosographic categories of online addiction and the resulting difficulties in describing the impact on users’ mental health, the present cross-sectional study aimed to gain new insights into the relationship between Internet addiction (especially social networking site [SNS] addiction), psychological distress, and physical discomfort, as well as gender differences in impact among users.

**Method:**

A sample of 583 Italian speakers (50.8% males; 48.7% females) with a mean age of 30.96 (SD = 12.12) completed an online survey in July 2021. A set of psychometric self-report instruments was administered to assess the study variables. Mediation analyses were performed for both the whole sample and across genders.

**Results:**

The study found that men exhibited higher levels of Internet addiction and craving than women, but no differences were found for SNS addiction. Furthermore, indicators of psychological distress (i.e., anxiety, depression, stress, loneliness, insomnia, and self-esteem) mediated the association between SNS addiction and body uneasiness, with slight differences across genders.

**Conclusion:**

This paper contributes to the existing literature on online addictive behaviors by also highlighting gender differences. The findings underscore the need for educational experiences that can prevent problematic use of the Internet and SNSs.

The Internet is widely available worldwide, and user access continues to grow. The Covid-19 pandemic has led to an increase in Internet connectivity in recent years, as it has been the only means of communication with others (Bhandari, 2020). In 2022, 69% of the world’s population and 89.7% of European inhabitants have been surfing the Internet (Internet World Stats, 2022). Currently, 6.3 billion people use smartphones to access the Internet, and this trend is expected to grow in the coming years, reaching 7.5 billion by 2026 (Statista, 2021). These digital devices provide simple access to the Internet with its vast information, novel ways to communicate, and several potentials to increase knowledge due to their ease of use and portability.

The increasing use of the Internet has been accompanied by scientific concerns about addictive behavior on the Internet. However, different authors have used various terms to refer to this problematic behavior. For example, *Compulsive Internet Use* (Black et al., 1999), *Pathological Internet Use* (Davis, 2001), and *Internet Addiction* (IA; Sherer, 1997) have been used to define online addictive behavior or *Problematic Mobile Phone Use* (Billieux et al., 2015). All these different terms seem to refer more or less to the same concept: A user who is so involved in online use that he/she neglects other areas of his/her life (Widyanto & Griffiths, 2006). However, the choice of a single designation seems premature, as the Diagnostic and Statistical Manual of Mental Disorder (DSM-5th text revised; American Psychiatric Association, 2022) has not yet come out clearly. Although many researchers have raised questions about the improvement of Internet addictive behaviors during the pandemic (Masaeli & Farhadi, 2021), some results have showed a similar occurrence compared to the pre-pandemic era (Burkauskas et al., 2022).

Despite the lack of terminological agreement, many studies (Ballarotto et al., 2021; Bottaro & Faraci, 2022; Cipolletta et al., 2020; Holmberg et al., 2018; Verduyn et al., 2020) have addressed the negative psychological effects of Internet use. Previous findings have shown that IA is a predictor of stress, depression, anxiety, insomnia, loneliness and low self-esteem (Corrêa Rangel et al., 2022; Mamun et al., 2020; Mathew & Krishnan, 2020; Saikia et al., 2019; Stanković et al., 2021; Younes et al., 2016; Yücens & Üzer, 2018) in both men and women (Ha & Hwang, 2014), although the results are still inconclusive. Thus, Costa et al. (2019) have shown that mechanisms for detecting satisfying social relationships rely on sensory information and body feedback; these cannot be exploited in online interactions, which have negative consequences in terms of loneliness. Therefore, loneliness is positively associated with IA (Mamun et al., 2020; Saadati et al., 2021; Simcharoen et al., 2018) and leads to social avoidance (Ahmed et al., 2021). However, a non-statistically significant association between loneliness and IA has been found in another research (Turan et al., 2020). In summary, further studies are needed to establish a possible causal relationship between IA and other relevant variables (e.g., loneliness, self-esteem, etc.). For example, Moretta and Buodo (2020) have recently suggested a dynamic relationship between IA and loneliness that could lead to a vicious cycle, with IA as a possible starting point.

On the other hand, according to recent studies (Ostovar et al., 2016), mood disorders in people with IA were a relevant outcome in about one in three patients (Ostinelli et al., 2021). Furthermore, the role of self-esteem in IA is unclear. While some studies focused on the predictive role of self-esteem (Aydm & San, 2011; Stieger & Burger, 2010), others pointed to a reciprocal relationship (Wang et al., 2022). However, most studies have investigated its mediating role in the association between IA and psychological well-being (Pichardo et al., 2021; Senol-Durak & Durak, 2011). In addition, a recent review and meta-analysis has highlighted the strong association between IA and sleep disorders (Alimoradi et al., 2019). In the current study, the focus is on the predictive role of IA for the indicators of psychological distress, which provides important insights into the health of online surfers (see Hypothesis 2).

Recently, the *Interaction of Person-Affect-Cognition-Execution Model* (I-PACE model; Brand et al., 2019) has been used to analyze the psychological and neurobiological processes common to addictive behaviors. The process model considers IA as “the consequence of interactions between predisposing factors, such as neurobiological and psychological constitutions, moderators, such as coping styles and Internet-related cognitive biases, and mediators, such as affective and cognitive responses to situational triggers in combination with reduced executive functioning. Conditioning processes may strengthen these associations within an addiction process” (Brand et al., 2016, p. 1). This theoretical model is a valuable starting point for the purpose of the present study. The subjectively perceived situation (e.g., stress, personal conflict, and abnormal mood) and the affective and cognitive response (e.g., urge for mood regulation, craving, and attentional bias) have been recognized as important constructs for understanding online addictions (see Hypothesis 3). The I-PACE model also points out that craving is the key mechanism for maintaining online addictive behaviors, with negative consequences in daily life, as well as substance addictions (Anton, 1999; Sun et al., 2021). Namely, craving is defined as an urgent and uncontrollable desire to use a substance or engage in a particular behavior (Caretti et al., 2008). Negative health outcomes of craving have been documented for various addictive behaviors (e.g., Joyner et al., 2015; King et al., 2016), but there do not appear to be any studies examining the direct effects of craving on psychological distress in Internet addiction, as far as we know. Furthermore, according to studies on addiction disorders, men progress more rapidly from behavior onset to dependence than women (Becker & Hu, 2008; Hallam et al., 2016).

Gender differences in Internet Addiction (IA) have been of interest in recent research. Some studies have shown that men experience higher levels of IA than women (Shan et al., 2021), with more intense experiences for men (Veltri et al., 2014) and worse psychological outcomes for women (Twenge & Martin, 2020). However, other studies have not found gender differences in IA (Shen et al., 2021). Gender differences have been found for several types of IA. For example, a recent meta-analysis (Su et al., 2020) has confirmed a gender-specific distinction, highlighting that men are more likely to suffer from Internet gaming disorder, whereas women are more likely to experience social networking site (SNS) addiction.

It is important to note that *internet addiction* is an umbrella term that encompasses different types of addictive online surfing behaviors, such as excessive shopping, gaming, and working. For the specific purpose of the present study, which mainly focuses on Social Networking Sites addiction (SNS), we will present the previous findings on this particular type of online behavior.

## Social Networking Sites Addiction

SNS addiction is a specific type of IA strongly associated with smartphone use (e.g., Instagram was developed only for smartphones; for a time, it was possible to use the platform on a PC as well, but with significant functional limitations) and body image (Ryding & Kuss, 2020). Kuss and Griffiths (2011, p. 3539) defined SNSs as “virtual communities where users can create individual public profiles, interact with real-life friends, and meet other people based on shared interests.” Although not yet defined as a diagnostic category with different theories and models (Sun & Zhang, 2021), the prevalence of SNS addiction is increasing in both men and women in different countries (Cheng et al., 2021), with a higher incidence in women than in men (Aparicio-Martínez et al., 2020; Su et al., 2020).

Social Networking Sites (SNS) addiction is associated with negative psychological symptoms such as anxiety, stress, depression (Hussain & Griffiths, 2018), and insomnia (Lin et al., 2021). Specifically, due to the interactive nature of SNSs, a recent study has showed that SNS users have higher levels of social appearance anxiety than individuals who do not use SNSs (Korkmazer et al., 2022). A recent meta-analysis (Zhang et al., 2022) has supported the association between SNS addiction and loneliness, with no differences between genders. In addition, some studies (Bányai et al., 2017; Busalim et al., 2019) have found an association between SNS addiction and self-esteem. The direction of this association is controversial. Many studies (Ahmed et al., 2021; Błachnio et al., 2016; Busalim et al., 2019; Seabra et al., 2017) have suggested that low self-esteem predicts SNS addiction in various countries such as Turkey, Italy, and the United States. However, other studies have shown a large impact of SNS addiction on self-esteem (for a review, see Krause et al., 2021). According to the *social compensation hypothesis* (Kraut et al., 2002), people who have low levels of self-esteem compensate for their social interaction problems with online “no-body” relationships (Lancini, 2015). The “like” mechanism typical of SNSs has a large negative impact on people with low levels of self-esteem (Martinez-Pecino & Garcia-Gavilan, 2019; Yücens & Üzer, 2018). A recent systematic review (Ryding & Kuss, 2020) has found that SNS addiction is a risk factor for body image dissatisfaction. According to the *social comparison theory* (Festinger, 1954), which also extends to online spaces characterized by perfect self-presentation, it has been suggested that people who overuse SNSs may decrease their self-acceptance (Cipolletta et al., 2020; Holmberg et al., 2018; Verduyn et al., 2020). These processes of self-comparison with others on SNSs reinforce users’ negative body perceptions. Excessive use of SNS, which includes sharing online photos and appearance-based comparisons, is often associated with control of body image in photos (Boursier et al., 2020; Ryding & Kuss, 2020), increases in psychological problems, such as depressive symptoms and weight control behaviors (Patte et al., 2021; Pollina-Pocallet et al., 2021; Zhang & Liu, 2021), and decreases in self-esteem (Andreassen et al., 2017).

Finally, body image concerns are associated with SNS addiction symptoms. According to Fardouly and Vartanian (2016), SNSs may contribute to body image concerns in several ways: i) SNSs are used by ‘regular people’ and not just celebrities, ii) people upload only the most attractive pictures of themselves, iii) peer comparison is common and could be more influential for the users’ body image, and iv) comments could influence self-perception. Moreover, these associations showed different negative psychological effects across genders (Boursier et al., 2020; Ryding & Kuss, 2020; Yurdagül et al., 2021). For example, problematic Instagram use (i.e., a popular SNS platform) “was directly associated with loneliness, general anxiety, and social anxiety among males only, whereas among females, problematic Instagram use was indirectly associated with general and social anxiety but was not related to loneliness” (Yurdagül et al., 2021, p. 1385). Further, body image uneasiness showed controversial results between genders: Some previous studies (Fischetti et al., 2020) reported that women were more dissatisfied than men, but other results showed the same relationship for men and women (Holland & Tiggemann, 2016). However, men appeared to engage in greater body advertising on SNS than women (Mahon & Hevey, 2021).

## Objectives and Hypotheses

The evidence to date suggests that online addictions (e.g., IA and SNS addiction) are not yet clearly defined and distinguishable nosographic categories. This has led to confounding results regarding the impact on users’ psychological well-being, including gender differences. Further evidence is needed to provide a clear definition of diagnosis and outcomes.

**Figure 1:**
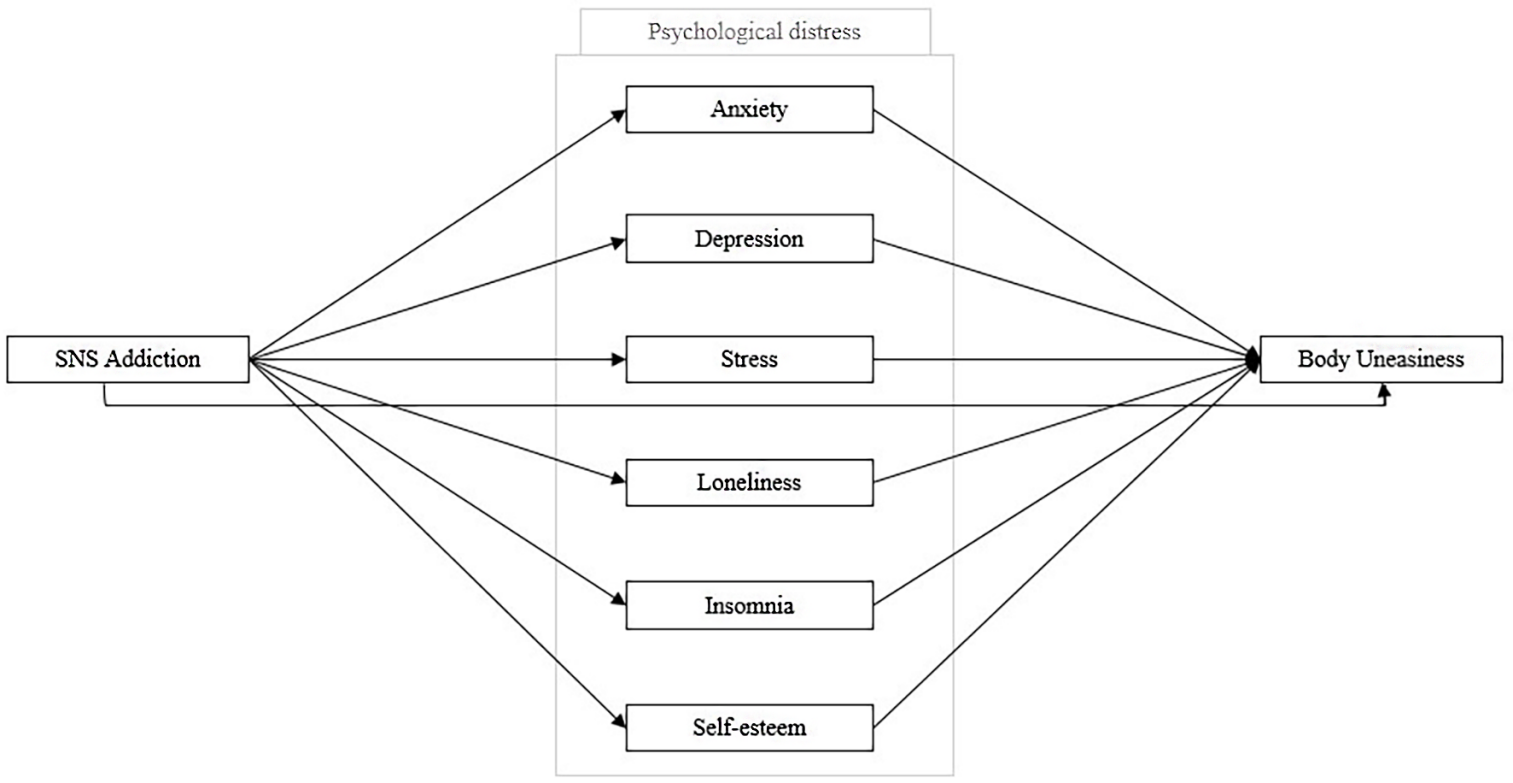
Hypothesized Mediation Model of Social Networking Sites Addiction, Psychological Distress, and Body Uneasiness *Note.* SNS Addiction = Social Networking Sites Addiction.

The present study aimed to: i) provide new insights into the relationship between Internet disorders and SNS addiction and its negative impact on psychological distress, ii) deliberately focus on a theoretical model of SNS addiction and its little-studied association with body uneasiness, and iii) assess gender differences. We also proposed a new theoretical model (Figure 1) to explain how SNS addiction might negatively affect users’ body uneasiness through indicators of psychological distress and tested it for both the whole sample and the two genders separately. This study may provide new insights into a previously undefined diagnostic category while having important clinical implications. Thanks to an exploratory gender perspective, it could make a significant contribution to the discovery of intermediate differences in the relationship with the Internet and SNS use.

Based on the above literature findings and the objectives of the present study, we derived the following research hypotheses:

Hypothesis 1 (H1): Internet addiction, craving, and SNS addiction are not equally distributed among men and women. Specifically, males are expected to report higher levels of Internet addiction and craving, while females will report higher levels of SNS addiction.

Hypothesis 2 (H2): Internet addiction and craving behavior will predict indicators of psychological distress (i.e., anxiety, depression, stress, loneliness, insomnia, and self-esteem) in both men and women.

Hypothesis 3 (H3): SNS addiction will show a positive association with body uneasiness.

Hypothesis 4 (H4): The association between SNS addiction and body uneasiness will be mediated by indicators of psychological distress (i.e., anxiety, depression, stress, loneliness, insomnia, and self-esteem).

Hypothesis 5 (H5): A similar pattern of relations between genders is expected, but with different magnitudes: greater direct and indirect effects for women than for men.

## Method

### Participants

The sample consisted of 583 Italian speakers living in Italy. Gender was balanced (296 males, 50.8%; 284 females, 48.7%), and the average age was 30.96 years (*SD* = 12.12). Only three participants preferred not to report their gender (0.5%). They mostly had an intermediate level of education (high school diploma = 42%; degree = 44.1%) and used SNS mainly to keep in touch with others (e.g., chatting and sharing photos or videos; 63.1%). Further descriptive information about the sample can be found in Table 1.

**Table 1:** Sample Descriptive Characteristics (N = 583)

Variable	*N*	%	Men		Women		I prefer not to specify	
			*N*	%	*N*	%	*N*	%
Educational level								
Middle License	29	5	18	6.1	11	3.9	0	0
High School Graduation	245	42	134	45.3	110	38.7	1	33.3
Degree	257	44.1	118	39.9	137	48.2	2	66.7
Post-degree	52	8.9	26	8.8	26	9.2	0	0
Greater use of Internet								
Stay with others (e.g., chat, share photos and videos)	369	63.3	169	57.1	198	69.7	2	69.7
Leisure (e.g., play)	133	22.8	83	28	50	17.6	0	0
Shopping online (e.g., Amazon, eBay)	42	7.2	21	7.1	21	7.4	0	0
Work	20	3.4	9	3.0	10	3.5	1	33.3
News	13	2.2	11	3.7	2	.7	0	0
Other	5	.9	3	1.0	2	.7	0	0

### Measures

The present study used ad hoc items to capture the sociodemographic characteristics of the sample. In addition, the following psychometric self-report instruments were used to assess the selected variables.

#### Internet Addiction

The Internet Addiction Test (IAT; Young, 1996, 1998) is the most commonly used psychometric instrument for the assessment of Internet addiction. It was developed according to the *Diagnostic and Statistical Manual of Mental Disorders* (4th edition) criteria for pathological gambling. The IAT consists of 20 items (e.g., “Do people around you complain about the amount of time you spend online?” and “Are your performance at work or your productivity affected negatively by the Internet?”) that assess the disorder’s severity on a 5-point Likert scale ranging from 1 (*not at all*) to 5 (*always*). In this study, we used its Italian version (Faraci et al., 2013), which also demonstrated good psychometric properties for the one-factor solution (Cronbach’s α = .91). The present sample’s Cronbach’s α was .94.

#### Craving

The Severity Index (Second Section) from the Addictive Behavior Questionnaire (ABQ_SI; Caretti et al., 2016), an Italian psychometric instrument, was selected to point out craving specific to Internet addiction. Unlike other craving tools, it does not refer to a particular type of addiction but has already been validated in Italy in reference to Internet addiction. Its 11 items (e.g., “I happen to think about the last time I went online” and “Thoughts and images related to Internet use suddenly emerge”) use a 5-point Likert scale ranging from 0 (*not at all*) to 4 (*always*). This instrument showed good reliability (Cronbach’s α = .84) and validity. The Cronbach’s α for the present sample was .82.

#### Social Networking Sites Addiction

The Bergen Social Media Addiction Scale (BSMAS; Andreassen et al., 2016) is a 6-item (e.g., “Have you tried to stop using social media without succeeding?” and “Have you become anxious or agitated if you’ve been banned from using social media?”) self-report scale that assesses addictive behaviors related to the use of SNSs. It measures six core features of social media addiction: salience, mood modification, tolerance, withdrawal, conflict, and relapse, and it uses a 5-point Likert scale ranging from 1 (*very rarely*) to 5 (*very often*). A high score is indicative of SNS addiction. Its Italian version (Monacis et al., 2017) confirmed the single-factor structure of the original instrument and showed good psychometric properties (Cronbach’s α = .88; Andreassen et al., 2016). For the present sample the Cronbach’s α was .87.

#### Self-Esteem

To evaluate the levels of self-esteem, we used the Rosenberg Self-esteem Scale (RSES; Rosenberg, 1965). RSES is a 10-item (e.g., “I think I have a number of qualities” and “I guess I don’t have much to be proud of”) self-report scale that uses a 4-point Likert scale ranging from 1 (*strongly agree*) to 4 (*strongly disagree*). We used its Italian version (Prezza et al., 1997), which showed good internal coherency (Cronbach’s α = .84). In the present study the Cronbach’s α for the RSES was .91.

#### Stress, Anxiety, and Depression

To assess stress, anxiety, and depression, we used the Depression Anxiety Stress Scales-21 (DASS-21; Lovibond et al., 1995) in its Italian version (Bottesi et al., 2015). It is a short measure that evaluates three indicators of psychological distress simultaneously. The 21-item (e.g., “I felt a lot of tension and I had difficulty recovering a state of calm,” “I just couldn’t feel any positive emotions,” and “I felt stressed out”) self-report scale uses a 4-point Likert scale ranging from 0 (*never happens to me*) to 3 (*it almost always happens to me*). Indeed, it has good internal consistency and temporal stability for each subscale (anxiety Cronbach’s α = .74; depression Cronbach’s α = .82; stress Cronbach’s α = .85). In the present sample all of the subscales showed good internal consistency (anxiety Cronbach’s α = .87; depression Cronbach’s α = .92; stress Cronbach’s α = .91).

#### Loneliness

The UCLA Loneliness Scale, Version 3 (UCLA-LS; Russell, 1996) was used to measure one’s subjective feelings of loneliness in its Italian version (Boffo, Mannarini, & Munari, 2012). The 20-item scale uses a 4-point Likert scale ranging from 1 (*I never feel this way*) to 4 (*I often feel this way*; e.g., “I am unhappy doing so many things alone” and “I feel isolated from others”). This measure had high internal consistency (Cronbach’s α = .96; Russell, 1996) and a test-retest correlation of .73 over 2 months. The Cronbach’s α for the present sample was .92.

#### Body Perception

The Body Uneasiness Test (BUT) is a self-report instrument to evaluate body perception that was first presented at the 5th International Conference on Eating Disorders (New York, 24–26 April 1998). In line with the objectives of the present study, we used its Italian version (Cuzzolaro et al., 2006), specifically Part A, the Global Severity Index (BUT_GSI). The BUT_GSI derives from the average rating of all 34 items constituting the BUT Part A, which is composed of five subscales (i.e., weight phobia, body image concerns, avoidance, compulsive self-monitoring, and depersonalization). The items use a 6-point Likert scale ranging from 0 (*not at all*) to 5 (*always*; e.g., “I spend a lot of time in front of the mirror” and “When I undress I avoid looking at myself”). It showed good internal coherency (Cronbach’s α ranged from .79 to .90 for each subscale). In the present sample, which used only the total score for BUT, the BUT-GSI showed a good Cronbach’s α = .97.

#### Insomnia

The Insomnia Severity Index (ISI; Morin et al., 2011), in its Italian version (Castronovo et al., 2016), was used to assess sleep quantity and quality. This 7-item self-report questionnaire assesses, during the prior 2 weeks, the severity of sleep onset, the severity of sleep maintenance, early morning awakenings, satisfaction level with the current sleep pattern, interference with daily living, noticeability of impairment due to the sleep difficulty, and level of distress caused by the sleep problem. The total score ranges from 0 to 28, with a higher score indicating greater insomnia severity. Each item is rated on a 5-point Likert scale ranging from 0 to 4 (for items 1–3, 0 = *no problem* and 4 = *very severe problem*; for item 4, 0 = *very satisfied* and 4 = *very dissatisfied*; for items 5–7, 0 = *not at all* and 4 = *very much*; e.g., “How satisfied/dissatisfied are you with your current sleep?”). The internal reliability coefficient was 0.75 (Morin et al., 2011). The Cronbach’s α for the present sample was .83.

### Procedure and Ethics

Data were collected in July 2021 through an online survey on SNSs (e.g., Facebook, Instagram, and WhatsApp; *N* = 244) and Amazon Mechanical Turk (*N* = 339). To avoid missing data, participants completed the online survey using a Google Form with a mandatory response format. According to our eligibility criteria, Italians older than 18 voluntarily participated in the research. After explaining the purposes of the study, all participants gave their informed consent.

The research project proposal was conducted in accordance with the Declaration of Helsinki and was approved by the Internal Review Board of the psychological research of the University Kore of Enna with the code UKE-IRBPSY-07.21.03. The administration of the measures was carried out in a fixed order and in compliance with the privacy guarantee regulations according to Legislative Decree n. 196/2003 and the GDPR (EU Regulation n.2016/679). On average, participants spent 20 minutes responding to the survey.

### Data Analyses

Using G*Power (Faul et al., 2007), we conducted a sensitivity power analysis to determine the minimum effect size required to find statistical significance. Performing a sensitivity power analysis is useful to see whether, given a limited *N*, the size of the effect that can be found is at all realistic (or, otherwise, too large to be expected realistically) (Faul et al., 2007). Given a sample size of 583, an alpha level of .05, and a minimum power of .80 (Cohen, 1992), there is an 80% chance of detecting an effect size of *f*^2^ = .02, assuming statistical significance and that such an effect size actually exists. Before testing our hypotheses, we first analyzed the correlations between all variables. We then performed an independent samples *t*-test to explore eventual differences in Internet addiction, craving, and SNS addiction across genders (H1). Additionally, we conducted a series of standard multiple regression analyses using the Statistical Package for Social Sciences Version 25 (SPSS v.25) to test H2 for both the total sample and gender groups. Further, we tested our theoretical model using the jAMM Module for Jamovi 2 (Gallucci, 2020; Jamovi Project, 2021; R Core Team, 2021; Rosseel, 2019; Soetaert, 2019) to analyze the association between SNS addiction and body uneasiness (direct effect; H3) and the mediation hypothesis (indirect effect) by psychological distress indicators (i.e., anxiety, depression, stress, loneliness, insomnia, and self-esteem) for the whole sample (H4) and across genders (H5).

## Results

### Preliminary Analyses

**Figure 2:**
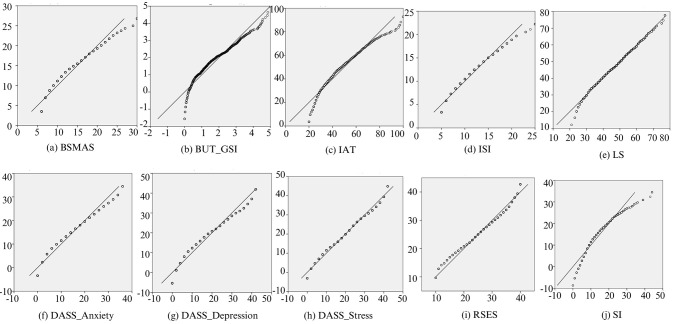
Q-Q Plots of the Study Variables *Note.* IAT = Internet Addiction Test; SI = Addictive Behavior Questionnaire Severity Index; BSMAS = Bergen Social Media Addiction Scale; DASS-21 = Depression, Anxiety, Stress Scale; LS = Loneliness Scale; ISI = Insomnia Severity Index; RSES = Rosenberg Self-Esteem Scale; BUT_GSI = Body Uneasiness Test_Global Severity Index.

The descriptive statistics (Table 2) highlighted the score distribution in the sample. The normality distribution of the investigated variables was checked through the inspection of both skewness and kurtosis statistics, which were regarded as indicative of a violation of the assumption of normality if > |1| and |3|, respectively (Kim, 2013). Results from our analyses indicated that, except for SI, which reported a skewness value greater than 1, all the other variables reported normality tests under the recommended thresholds, suggesting that they were normally distributed. Figure 2 depicts the graphical visualization (i.e., Q-Q plots) of the variables’ distribution. Moreover, moderate to high positive correlations among the study variables were found (estimated at *p* < .001), except for self-esteem, which reported negative associations (*p* < .001; Table 3).

**Table 2:** Descriptive Statistics for the Study Variables

Variable	Sample		Men		Women		Skewness	Kurtosis
	*M*	*SD*	*M*	*SD*	*M*	*SD*		
IAT	46.9	16.6	50.1	16.8	43.6	15.7	0.80	0.42
SI	13.3	7.62	14.7	8.0	12.0	7.0	1.21	1.88
BSMAS	12.9	5.67	13.2	5.7	12.7	5.6	0.86	0.19
DASS-21_Anxiety	12.0	9.32	11.5	8.8	12.6	9.7	0.63	-0.43
DASS-21_Depression	16.3	11.8	16.3	11.1	16.3	12.4	0.51	-0.73
DASS-21_Stress	20.4	10.9	19.8	10.2	21.0	11.5	0.07	-0.75
LS	45.9	11.0	47.5	10.7	44.3	11.2	0.18	-0.39
ISI	11.0	4.17	11.2	4.2	10.9	4.1	0.70	0.24
RSES	29.4	7.38	29.5	7.3	29.4	7.4	-0.51	-0.45
BUT_Global Severity Index	1.54	1.12	1.5	1.1	1.6	1.1	0.77	-0.10

*Note.* IAT = Internet Addiction Test; SI = Addictive Behavior Questionnaire Severity Index; BSMAS = Bergen Social Media Addiction Scale; DASS-21 = Depression, Anxiety, Stress Scale; LS = Loneliness Scale; ISI = Insomnia Severity Index; RSES = Rosenberg Self-Esteem Scale; BUT = Body Uneasiness Test.

**Table 3:** Correlations Between the Study Variables

Variable	1	2	3	4	5	6	7	8	9	10
1. IAT	—									
2. SI	.84***	—								
3. BSMAS	.82***	.77***	—							
4. DASS-21_Anxiety	.49***	.48***	.52***	—						
5. DASS-21_Depression	.50***	.48***	.54***	.75***	—					
6. DASS-21_Stress	.43***	.40***	.49***	.78***	.81***	—				
7. LS	.45***	.45***	.43***	.43***	.63***	.48***	—			
8. ISI	.35***	.32***	.36***	.46***	.44***	.42***	.38***	—		
9. RSES	-.48***	-.45***	-.48***	-.51***	-.63***	-.50***	-.51***	-.31***	—	
10. BUT_Global Severity Index	.57***	.56***	.56***	.57***	.62***	.52***	.51***	.42***	-.58***	—

*Note.* IAT = Internet Addiction Test; SI = Addictive Behavior Questionnaire Severity Index; BSMAS = Bergen Social Media Addiction Scale; DASS-21 = Depression, Anxiety, Stress Scale; LS = Loneliness Scale; ISI = Insomnia Severity Index; RSES = Rosenberg Self-Esteem Scale; BUT = Body Uneasiness Test.

***p < .001.

### Gender Differences in Internet Addiction, Social Networking Sites Addiction, and Craving

To test H1, we performed an independent samples *t*-test for IA, craving, and SNS addiction (Table 4). The results showed a significant gender difference for IA (*t* = 4.76, *p* < .001, Cohen’s *d* = .395) and craving (*t* = 4.36, *p* < .001, Cohen’s *d* = .362) (i.e., men showed higher levels of Internet addiction and craving than women), but no difference for SNS addiction (*p* = .285).

**Table 4:** Results of Independent Samples t-Test for Men and Women

Variable	Student’s *t*	*df*	*p*	Cohen’s *d*	95% CI	
					*LL*	*UL*
IAT	4.76	578	< .001	.395	0.23	0.56
SI	4.36	578	< .001	.362	0.20	0.53
BSMAS	1.07	578	.285	.089	-0.07	0.25

*Note.* IAT = Internet Addiction Test; SI = Addictive Behavior Questionnaire Severity Index; BSMAS = Bergen Social Media Addiction Scale; CI = Confidence interval; *LL* = Lower limit; *UL* = Upper limit.

### Internet Addiction, Craving, and Psychological Distress

Before testing H2, we evaluated the collinearity between IA and craving. The collinearity diagnostic results did not show any problematic values (Tolerance = 0.29, Variance Inflaction Factor = 3.39, and Condition Index < 15). We performed several multiple regression analyses in which IA and craving were entered as predictors, and the psychological distress indicators (i.e., stress, anxiety, depression, loneliness, self-esteem, and insomnia) were specified as criterion variables for both the whole sample and disaggregated by gender. Generally, for the whole sample, both IA and craving predicted the selected psychological distress indicators. High levels of IA predicted high levels of psychological distress (anxiety: β = 0.16, *t* = .44, *p* < .001; depression: β = 0.24, *t* = 5.10, *p* < .001; stress: β = 0.21, *t* = 4.60, *p* < .001; loneliness: β = 0.15, *t* = 3.45, *p* < .001; insomnia: β = 0.07, *t* = 3.79, *p* < .001), but also low levels of self-esteem (β = -0.16, *t* = -5.27, *p* < .001). Likewise, high levels of craving predicted high levels of psychological distress (anxiety: β = 0.29, *t* = 3.63, *p* < .001; depression: β = 0.31, *t* = 3.07, *p* < .01; stress: β = 0.20, *t* = 2.02, *p* < .05; loneliness: β = 0.38, *t* = 3.90, *p* < .001), except for insomnia (β = 0.05, *t* = 1.35, *p* = .178), and low levels of self-esteem (β = -0.14, *t* = -2.25, *p* < .05).

Secondly, the standard multiple regression analyses disaggregated by gender (Table 5) supported the previous findings also for men, confirming IA and craving as predictors for most psychological distress indicators. However, IA was not able to predict loneliness (β = 0.05, *t* = .48, *p* = .629), and craving was not able to predict insomnia for men (β = 0.15, *t* = 1.55, *p* =.121). On the other hand, IA was able to predict all the investigated indicators of psychological distress, but craving did not predict any psychological distress indicators for women.

**Table 5:** Regressions of Associations Between Internet Addiction, Craving, and Psychological Distress Indicators Across Gender

Variable	Men				Women			
	β	*SE*	*t*	*p*	β	*SE*	*t*	*p*
Anxiety								
IAT	.34	.04	3.98	.000	.29	.06	3.09	.002
SI	.29	.09	3.40	.001	.18	.13	1.91	.057
Depression								
IAT	.34	.06	3.82	.000	.36	.07	3.90	.000
SI	.27	.12	3.06	.002	.13	.16	1.36	.174
Stress								
IAT	.29	.06	3.05	.002	.36	.07	3.81	.000
SI	.20	.12	2.12	.035	.10	.16	1.07	.285
Loneliness								
IAT	.05	.06	.48	.629	.40	.07	4.29	.000
SI	.43	.13	4.55	.000	.08	.15	.89	.373
Insomnia								
IAT	.31	.02	3.18	.002	.25	.03	2.45	.015
SI	.15	.05	1.55	.121	.01	.06	.07	.944
Self-esteem								
IAT	-.28	.04	-3.01	.003	-.46	.04	-4.92	.000
SI	-.27	.08	-2.98	.003	-.02	.10	-.19	.846

*Note.* IAT = Internet Addiction Test; SI = Addictive Behavior Questionnaire Severity Index.

### The Proposed Mediation Model: Associations Between Social Networking Sites Addiction, Body Uneasiness, and Psychological Distress Indicators as Mediators

**Figure 3:**
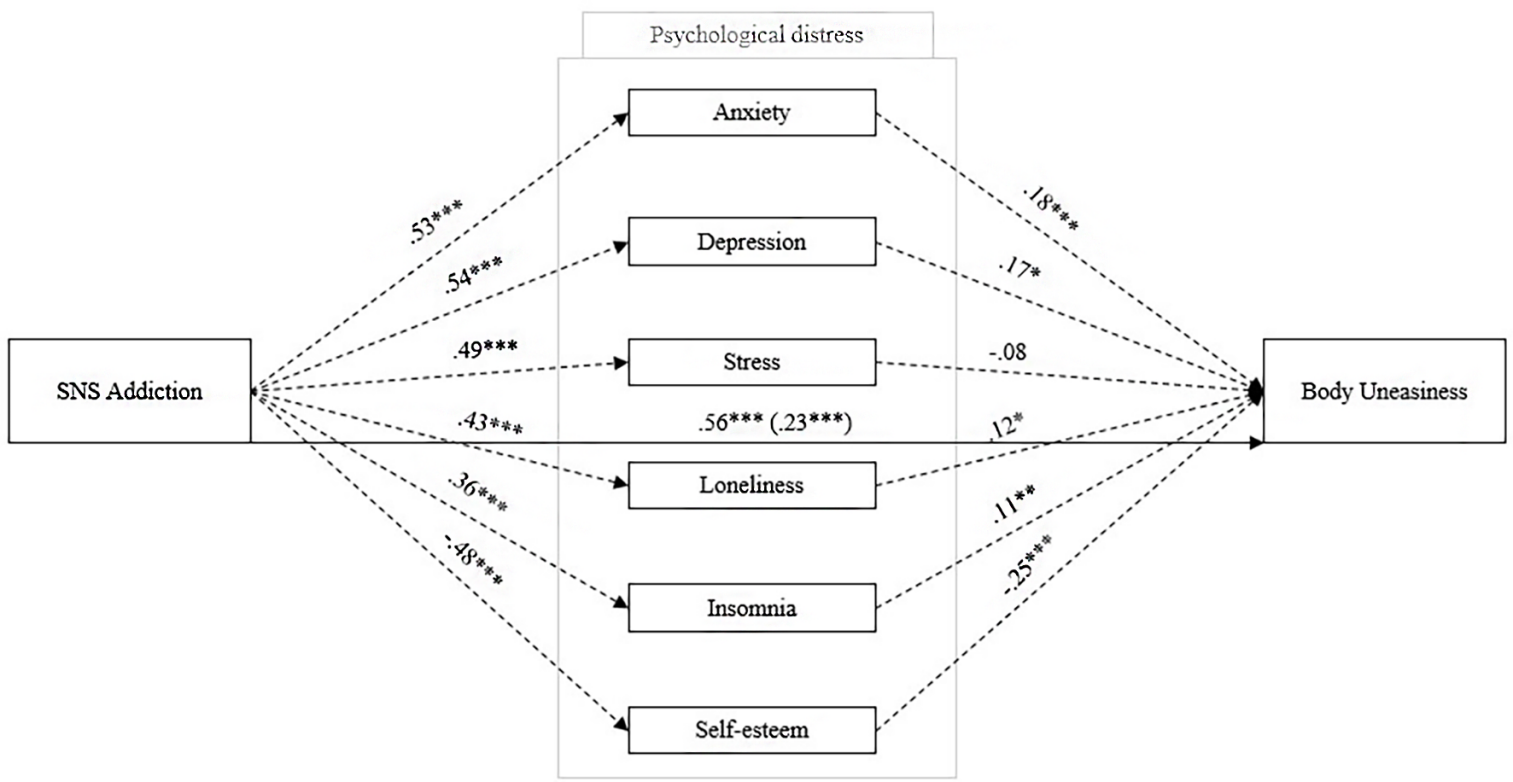
Measurement Model Testing the Relationship Between Social Networking Sites Addiction and Body Uneasiness Mediated by Psychological Distress for the Whole Sample *Note.* Coefficients are standardized betas; dotted lines indicate components; solid lines indicate total and direct effects (direct effect are shown in parentheses). *p < .05. **p < .01. ***p < .001.

Table 6 shows the results of the proposed model (Figure 1)—that is, when SNS Addiction is an independent variable, body uneasiness is a dependent variable, and psychological distress indicators are mediators. The use of a percentile bootstrap (Alfons et al., 2022) ensured robust statistical analysis. The direct effect (H2) showed significant results (β = 0.044, *p* < .001, 95% CI [0.098, 0.124]) and the magnitude of this effect increased when psychological distress indicators were introduced as mediators (H3; total effect: β = 0.111, *p* < .001, 95% CI [0.028, 0.059]). SNS addiction indirectly predicted body uneasiness through almost all psychological distress indicators (i.e., anxiety: β = 0.018, *p* < .001, 95% CI [0.007, 0.029]; depression: β = 0.018, *p* < .05, 95% CI [0.003, 0.032]; loneliness: β = 0.010, *p* < .01, 95% CI [0.003, 0.017]; insomnia: β = 0.038, *p* < .05, 95% CI [0.002, 0.013]; self-esteem: β = 0.022, *p* < .001, 95% CI [0.013, 0.032], respectively), explaining 61% of the total variance (anxiety 16%, depression 16%, loneliness 9%, insomnia 6%, and self-esteem 20%). However, when stress mediated the association between SNS addiction and body uneasiness, there was not a significant indirect effect, β = -0.007, *p* = 177, 95% CI [-0.019, 0.003] (Figure 3).

**Table 6:** Total, Direct, and Indirect Effects of the Hypothesized Mediation Model for SNS Addiction, Body Uneasiness, and Psychological Distress for the Whole Sample

Effect	Estimate	*SE*	95% CI		β	*z*	*p*
			*LL*	*UL*			
Indirect							
BSMAS ⇒ DASS21_Anxiety ⇒ BUT_GSI	0.018	0.005	0.007	0.029	0.095	3.19	< .001
BSMAS ⇒ DASS21_Depression ⇒ BUT_GSI	0.018	0.007	0.003	0.032	0.095	2.35	< .05
BSMAS ⇒ DASS21_Stress ⇒ BUT_GSI	0.007	0.005	-0.019	0.003	-0.040	-1.35	.177
BSMAS ⇒ LS ⇒ BUT_GSI	0.010	0.003	0.003	0.017	0.054	2.91	< .05
BSMAS ⇒ ISI ⇒ BUT_GSI	0.007	0.003	0.002	0.013	0.038	2.40	< .05
BSMAS ⇒ RSES ⇒ BUT_GSI	0.022	0.005	0.013	0.032	0.118	4.65	< .001
Direct							
BSMAS ⇒ BUT_GSI	0.044	0.008	0.028	0.059	0.023	5.58	< .001
Total							
BSMAS ⇒ BUT_GSI	0.111	0.007	0.098	0.124	0.563	16.43	< .001

*Note.* Confidence intervals computed with method: Bootstrap percentiles. Betas are completely standardized effect sizes. CI = confidence interval; *LL* = lower limit; *UL* = Upper limit; BSMAS = Bergen Social Media Addiction Scale; DASS-21 = Depression, Anxiety, Stress Scale; LS = Loneliness Scale; ISI = Insomnia Severity Index; RSES = Rosenberg Self-Esteem Scale; BUT_GSI= Body Uneasiness Test_Global Severity Index.

### The Proposed Mediation Model Across Gender

**Figure 4:**
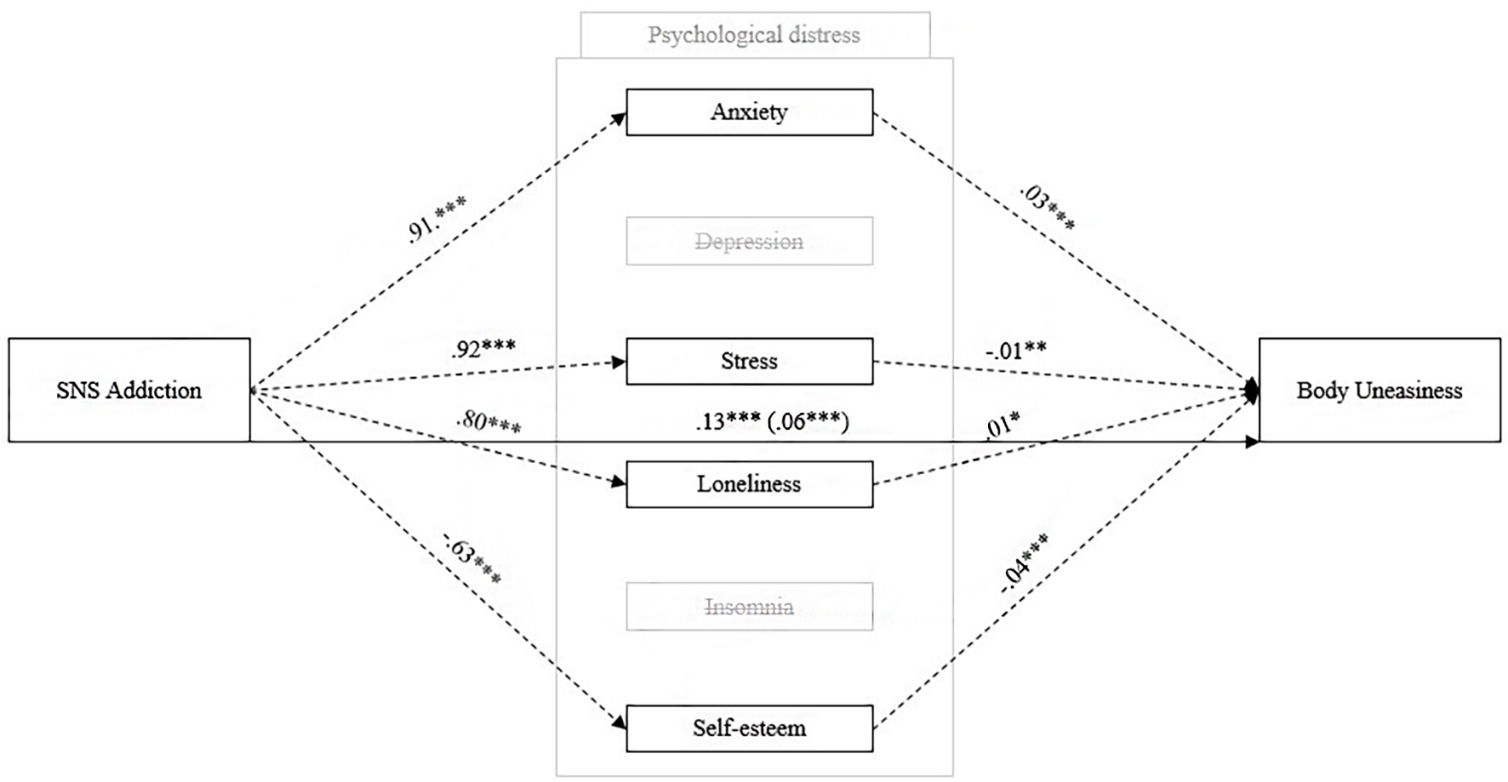
Measurement Model Testing the Relationship Between Social Networking Sites Addiction and Body Uneasiness Mediated by Psychological Distress for Men *Note.* Coefficients are standardized betas; dotted lines indicate components; solid lines indicate total and direct effects (direct effect are shown in parentheses); only significant results. *p < .05. **p < .01. ***p < .001.

Finally, we tested the proposed mediation model (Figure 1) across gender to test H4. Table 7 and Figure 4 show the results for men. The direct effect showed significant results, β = 0.063, *p* < .001, 95% CI [0.098, 0.124], and the magnitude of this effect increased when psychological distress indicators were introduced as mediators (total effect: β = 0.127, *p* < .001, 95% CI [0.028, 0.059]). SNS addiction indirectly predicted body uneasiness through almost all psychological distress indicators (i.e., anxiety: β = 0.025, *p* < .001, 95% CI [0.013, 0.036]; stress: β = -0.011, *p* < .05, 95% CI [-0.019, -0.002]; loneliness: β = 0.010, *p* < .01, 95% CI [0.003, 0.018]; insomnia: β = 0.006, *p* < .05, 95% CI [-0.001, 0.014], self-esteem: β = 0.025, *p* < .001, 95% CI [0.016, 0.035], respectively). However, when depression was entered as a mediator, there was no significant indirect effect, β = 0.008, *p* = 136, 95% CI [-0.002, 0.018].

**Table 7:** Total, Direct, and Indirect Effects of the Hypothesized Mediation Model for SNS Addiction, Body Uneasiness, and Psychological Distress for Men

Effect	Estimate	*SE*	95% CI		β	*z*	*p*
			*LL*	*UL*			
Indirect							
BSMAS ⇒ DASS21_Anxiety ⇒ BUT_GSI	0.025	0.006	0.013	0.036	0.129	4.28	< .001
BSMAS ⇒ DASS21_Depression ⇒ BUT_GSI	0.008	0.005	-0.002	0.018	0.041	1.49	.139
BSMAS ⇒ DASS21_Stress ⇒ BUT_GSI	-0.011	0.004	-0.019	-0.002	-0.055	-2.34	< .05
BSMAS ⇒ LS ⇒ BUT_GSI	0.010	0.004	0.003	0.018	0.053	2.72	< .01
BSMAS ⇒ ISI ⇒ BUT_GSI	0.006	0.004	-0.001	0.014	0.032	1.57	.116
BSMAS ⇒ RSES ⇒ BUT_GSI	0.025	0.005	0.016	0.035	0.132	5.11	< .001
Direct							
BSMAS ⇒ BUT_GSI	0.063	0.013	0.037	0.089	0.329	4.74	< .001
Total							
BSMAS ⇒ BUT_GSI	0.127	0.009	0.110	.0144	0.638	14.22	< .001

*Note.* Confidence intervals computed with method: Bootstrap percentiles. Betas are completely standardized effect sizes. CI = confidence interval; *LL* = Lower Limit; *UL* = Upper Limit; BSMAS = Bergen Social Media Addiction Scale; DASS-21 = Depression, Anxiety, Stress Scale; LS = Loneliness Scale; ISI = Insomnia Severity Index; RSES = Rosenberg Self-Esteem Scale; BUT_GSI= Body Uneasiness Test_Global Severity Index.

**Figure 5:**
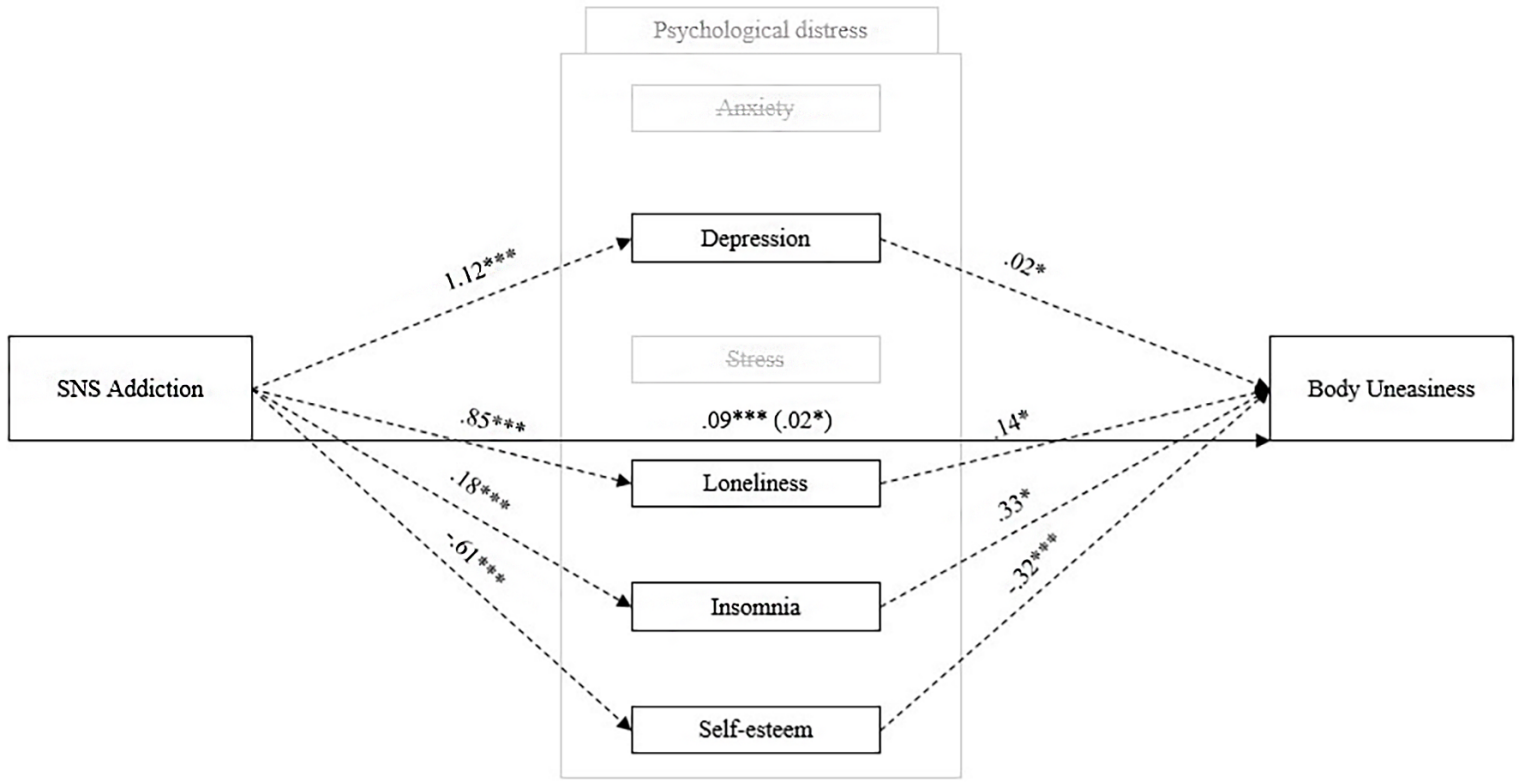
Measurement Model Testing the Relationship Between Social Networking Sites Addiction and Body Uneasiness Mediated by Psychological Distress for Women *Note.* Coefficients are standardized betas; dotted lines indicate components; solid lines indicate total and direct effects (direct effect are shown in parentheses); only significant results. *p < .05. ***p < .001.

The proposed model was also tested for women (Table 8, Figure 5). The direct effect showed a lower significant result (β = 0.026, *p* < .05, 95% CI [1.28e^-4^, 0.052]) than men, and the magnitude of this effect considerably increased when psychological distress indicators were introduced as mediators (total effect: β = 0.095, *p* < .001, 95% CI [0.075, 0.115]).

**Table 8:** Total, Direct, and Indirect Effects of the Hypothesized Mediation Model for SNS Addiction, Body Uneasiness, and Psychological Distress for Women

Effect	Estimate	*SE*	95% CI		β	*z*	*p*
			*LL*	*UL*			
Indirect							
BSMAS ⇒ DASS21_Anxiety ⇒ BUT_GSI	0.007	0.004	-0.002	0.016	0.038	1.580	.114
BSMAS ⇒ DASS21_Depression ⇒ BUT_GSI	0.028	0.006	0.0117	0.039	0.154	4.931	< .001
BSMAS ⇒ DASS21_Stress ⇒ BUT_GSI	-0.004	0.004	-0.013	0.005	-0.022	-0.901	.367
BSMAS ⇒ LS ⇒ BUT_GSI	0.012	0.004	0.004	0.021	0.067	2.896	< .05
BSMAS ⇒ ISI ⇒ BUT_GSI	0.006	0.002	0.001	0.011	0.034	2.390	< .05
BSMAS ⇒ RSES ⇒ BUT_GSI	0.020	0.005	0.010	0.029	0.108	4.006	< .001
Direct							
BSMAS ⇒ BUT_GSI	0.026	0.013	1.28e^-4^	0.052	0.141	1.970	< .05
Total							
BSMAS ⇒ BUT_GSI	0.095	0.010	0.075	0.115	0.487	9.374	< .001

*Note.* Confidence intervals computed with method: Bootstrap percentiles. Betas are completely standardized effect sizes. CI = confidence interval; *LL* = Lower Limit; *UL* = Upper Limit; BSMAS = Bergen Social Media Addiction Scale; DASS-21 = Depression, Anxiety, Stress Scale; LS = Loneliness Scale; ISI = Insomnia Severity Index; RSES = Rosenberg Self-Esteem Scale; BUT_GSI= Body Uneasiness Test_Global Severity Index.

SNS addiction indirectly predicted body uneasiness through almost all of the psychological distress indicators (i.e., depression: β = 0.028, *p* < .001, 95% CI [0.017, 0.039]; loneliness: β = 0.012, *p* < .01, 95% CI [0.004, 0.021]; insomnia: β = 0.006, *p* < .05, 95% CI [0.001, 0.011], self-esteem: β = 0.019, *p* < .001, 95% CI [0.010, 0.029], respectively). However, neither anxiety (β = 0.007, *p* = .114, 95% CI [-0.002, 0.016]) nor stress (β = -0.004, *p* = .367, 95% CI [-0.015, 0.005]) mediated the association between SNS addiction and body uneasiness.

## Discussion

The widespread use of Internet surfing and increasingly easy access to the Internet have led to greater interest in online addictive behaviors and their negative effects on users’ psychological well-being (Corrêa Rangel et al., 2022; Mamun et al., 2020; Mathew & Krishnan, 2020; Saikia et al., 2019; Stanković et al., 2021; Younes et al., 2016; Yücens & Üzer, 2018). However, the diagnostic criteria for online addictions are still not clearly shared. This has led to conflicting and confusing measurements and results. With the present study, we aimed to: (i) provide new insights into the gender differences in IA and craving in predicting indicators of psychological unwellness; (ii) propose a mediation model for the unexplored relationship between SNS addiction, indicators of psychological unwellness, and body uneasiness; and (iii) explore its gender differences. As suggested in previous research, our preliminary analyses supported the idea that IA is an umbrella term (Ryding & Kuss, 2020). In particular, the strong positive correlation analyses supported the theoretical conceptualization of SNS addiction as a distinct form of IA. In addition, previous studies (Su et al., 2020; Twenge & Martin, 2020) have found several gender differences in online addictive behaviors. Regarding H1, our results partially supported previous findings: Males in our sample were found to have higher levels of IA and craving. Based on our results, men―who more often use the Internet to play than women―show higher levels of addictive behavior and craving than women. While women’s presence in online games continues to increase, for men it is often a fun and intense behavior, they are more motivated to play, start earlier, and spend more time than women (Jung et al., 2020, Veltri et al., 2014). However, in contrast to previous research, SNS addiction did not show significant differences between genders. One possible explanation is that recently—also due to COVID-19 restrictions (Valenti & Faraci, 2021)—both men and women have seen SNS as an environment where they can express themselves and socialize beyond their physical presence. People may have experienced social networks as the only possible venue for communication (Bhandari, 2020), thus eliminating the gender differences seen prior to the pandemic. This collective experience may have made the pre- and post-lockdown results regarding the use of SNS not comparable.

Consistent with previous studies (Corrêa Rangel et al., 2022; Ha & Hwang, 2014; Krause et al., 2021; Mamun et al., 2020; Mathew & Krishnan, 2020; Saikia et al., 2019; Stanković et al., 2021; Younes et al., 2016; Yücens & Üzer, 2018) and H2, our results confirmed the negative effects of IA and craving on the selected indicators of psychological distress for both men and women. In particular, high scores of IA and craving predicted high scores of anxiety, depression, stress, loneliness, and low scores of self-esteem across the whole sample. Only insomnia was predicted by IA, not by craving. A possible explanation is that insomnia could also be a consequence of craving, as the desire to stay connected would lead to a reduction in sleep hours, likely resulting in a bidirectional relationship. However, this goes beyond the scope of this work. As a further confirmation, in gender-disaggregated analyses, IA predicted psychological distress indicators for both men and women. Only for men, IA was unable to predict loneliness. Based on these findings, women—who use the Internet more than men to stay connected with others―suffer from negative affect caused by a lack of social interaction, while men―who use the Internet less than women for interactions―experience few moments of online socialization. Men’s interpersonal relationships may not depend on the online world, and the consequences of IA in terms of loneliness are lower. A plausible explanation might be that men are more reluctant than women to admit their loneliness, especially when using psychometric self-report instruments, because of social masculine norms of independence and self-confidence. As a clinical implication, this knowledge could promote the use of targeted clinical treatments differentiated by gender to better tailor a patient-centered approach. In contrast to H2, craving predicted the indicators of psychological distress only in men. To our knowledge, there is no previous research on gender differences in Internet-related craving. Following controversial findings in the addiction literature, men show addictive behavior faster than women and report more intensive use from the beginning. As a possible explanation, the low average age of our sample might better describe the situation of individuals with relatively young addictive behaviors. Further studies, including longitudinal methods, should delve into the consequences of Internet craving disaggregated by gender. They should also reveal comparisons between different age groups or the age of first addictive use of the Internet. These findings could reveal different clinical treatments depending on the duration of IA and the severity of craving.

### The Association Between SNS Addiction and Body Uneasiness Mediated by Psychological Distress

We proposed a mediation model (Figure 1) to evaluate the association between SNS addiction and body uneasiness, where psychological distress mediated this association. Our results partially supported H3–H4 and were consistent with previous research (Boursier et al., 2020; Cipolletta et al., 2020; Holmberg et al., 2018; Krause et al., 2021; Patte et al., 2021; Pollina-Pocallet et al., 2021; Ryding & Kuss, 2020; Verduyn et al., 2020; Zhang & Liu, 2021). We found a partial indirect effect of SNS addiction on body uneasiness, when the psychological distress indicators were entered as mediators. According to our results and previous studies (Boursier et al., 2020; Ryding & Kuss, 2020; Pollina-Pocallet et al., 2021; Patte et al., 2021; Zhang & Liu, 2021), SNS addiction is partially able to predict body uneasiness, and the introduction of the psychological distress indicators as mediators provides a deeper understanding of the phenomenon. People might find online satisfaction of their social needs, such as recognition and approval, on SNS platforms through the like-mechanism. Based on this reasoning and according to the social compensation hypothesis (Kraut et al., 2002), psychological distress is a negative consequence when social recognition is not provided through the online interactions of others. As with any other addictions (Corrêa Rangel et al., 2022; Ha & Hwang, 2014; Mamun et al., 2020; Mathew & Krishnan, 2020; Saikia et al., 2019; Stanković et al., 2021; Younes et al., 2016; Yücens & Üzer, 2018), SNS addiction predicted poorer psychological well-being among users, with an increase in anxiety, depression, loneliness, and insomnia scores, and a decrease in self-esteem scores. As a clinical implication, prevention against intentional SNS reuse could be useful to provide opportunities for socialization in body-related contexts and to reduce time spent online. In addition, our findings showed a significant mediating role for psychological distress in the association between SNS addiction and body uneasiness. Based on social comparison theory (Festinger, 1954) extended to online platforms, SNS users frequently compare themselves to unattainable models. Based on these findings, the perception of one’s own body is worse when the level of SNS addiction increases, with adverse consequences for psychological distress. One possible interpretation is that individuals with higher levels of SNS addiction (associated with psychological distress) continue to be exposed to more harmful stimuli for their self-representation; constant comparison with perfect models would increase discomfort with one’s own body. Lastly, only stress showed a non-significant association with body uneasiness but was directly predicted by SNS addiction. One possible interpretation is that stress could be a consequence of SNS addictive behavior but does not affect body image because the world of SNSs ends not only in social comparison with others but also in addictive behaviors, that go beyond the body dimension (e.g., sports, news, politics, etc.). Further research should delve into the role of stress as a risk factor for online addiction.

Finally, we tested our proposed model across genders. Consistent with H4 and previous findings (Holland & Tiggemann, 2016), the overall results are similar for both the whole sample and the two subsamples disaggregated by gender. However, the direct effect of SNS addiction on body uneasiness (i.e., excluding the mediation of psychological distress) is higher for men than for women. One possible explanation is that men engage in more body-related behaviors in online spaces than women, which is also consistent with previous findings. Therefore, their body uneasiness might be more influenced by external feedback (e.g., the like mechanism) than by internal experiences of psychological distress. Moreover, we found some small differences regarding the influence of mediators: Specifically, depression and insomnia had no significant influences on men, whereas anxiety and stress had no significant influences on women. Although these results are rather prodromal in psychological research, they have some clinical implications: Psychological support for patients with body image disorders should also be provided by understanding their online lives (i.e., self-presentation and communication on SNS) to better understand the role of their psychological symptoms. Again, gender differences should be taken into account for an increasingly patient-tailored approach.

### Limitations

The results of the present work should be taken with caution because of some limitations. First, we examined the association between many variables, and second, our convenience sample was not very large and was recruited from two different sources (i.e., SNSs and Amazon MTurk). Further research should replicate the present findings with a better and larger sampling procedure and in other cultures. Finally, only some participants have received a small symbolic economic reward through Amazon Mechanical Turk (i.e., 0.5$); this could be a limit to the accuracy of the answers provided. Additional studies could examine whether the reward mechanism may have led to differences between groups.

### Conclusion

Despite its limitations, this empirical study provides new insight into IA and craving and their effects on psychological well-being, highlighting gender differences as well. Moreover, the proposed model provides a deeper understanding of the impact of SNS on body uneasiness, emphasizing the mediating role of psychological stress indicators across genders. At the theoretical level, our findings offer new perspectives for a better knowledge of the diagnostic classifications (i.e., IA and SNS addiction), which are not yet well defined, and their impact on users’ well-being. At the clinical level, the worst mental health consequences of Internet and SNS addiction should be addressed in programs guided by scientific evidence on other addictive behaviors and taking into account specific gender differences. As practical implications, we support measures to prevent excessive Internet and SNS use (Groth et al., 2017)—for example, schools, families, and informal educational agencies should activate teachable opportunities to learn healthy use of the Internet and prevent social discomfort and poor relationships with one’s own body. It is also important to look at long-term negative psychological symptoms to understand longitudinally how men and women cope with the Internet and SNS use to buffer the consequences of an increasingly pervasive technology. Overall, this research contributes to our understanding of addiction in the digital age and provides empirical findings that have direct implications for clinical practice and research (for example, by providing a better understanding of new addictions and contributing to their improved nosographic classification). It also highlights the need for continued efforts to promote healthy online behaviors. Indeed, these findings underscore the importance of focusing on users’ overall mental and physical health. The body and mind should be clinically cared for in a holistic manner.
